# Supplementary material for: An open-source framework for large-scale, flexible evaluation of biomedical text mining systems
Source: J Biomed Discov Collab. 2008 Jan 29;3:1. doi: 10.1186/1747-5333-3-1 (PMC2276192; doi:10.1186/1747-5333-3-1)
Supplement: Additional file 1 — Evaluation platform source code. The additional file contains the software evaluation framework discussed in this paper. The most current release can be downloaded from SourceForge.net [1]. See the accompanying README file for instructions on installation and use. Also included in the distribution is a collection of UIMA wrappers for some commonly used BioNLP tools and annotated corpora. [file 1747-5333-3-1-S1.zip › bionlp_uima_repository-1.0.1/docs/api/src-html/edu/uchsc/ccp/util/nlp/document/DocumentUtil.html]

```
001    package edu.uchsc.ccp.util.nlp.document;
002    
003    import java.util.HashMap;
004    
005    public class DocumentUtil {
006    
007    
008        public static HashMap<Integer, String> getSectionIDNameHash() {
009            HashMap<Integer, String> idNameHash = new HashMap<Integer, String>();
010            idNameHash.put(DocumentSectionTypes.OTHER_ID, DocumentSectionTypes.OTHER_NAME);
011            idNameHash.put(DocumentSectionTypes.TITLE_ID, DocumentSectionTypes.TITLE_NAME);
012            idNameHash.put(DocumentSectionTypes.ABSTRACT_ID, DocumentSectionTypes.ABSTRACT_NAME);
013            return idNameHash;
014        }
015        
016        public static HashMap<String, Integer> getSectionNameIDHash() {
017            HashMap<String, Integer> nameIDHash = new HashMap<String, Integer>();
018            nameIDHash.put(DocumentSectionTypes.OTHER_NAME, DocumentSectionTypes.OTHER_ID);
019            nameIDHash.put(DocumentSectionTypes.TITLE_NAME, DocumentSectionTypes.TITLE_ID);
020            nameIDHash.put(DocumentSectionTypes.ABSTRACT_NAME, DocumentSectionTypes.ABSTRACT_ID);
021            return nameIDHash;
022        }
023    
024    }
```
